# Short stature and melanocytic nevi in a girl with *ARID1B-*related Coffin-Siris syndrome: a case report

**DOI:** 10.1186/s12887-022-03535-4

**Published:** 2022-08-13

**Authors:** Dong-Ying Tao, Huan-Hong Niu, Jing-Jing Zhang, Hui-Qin Zhang, Ming-Hua Zeng, Sheng-Quan Cheng

**Affiliations:** 1 Department of Pediatrics, Xijing Hospital, Fourth Military Medical University, No. 127 Changle West Road, Xincheng District, Xi’an, 710032 Shaanxi Province China; 2 Medical Experiment and Training Center, Hanzhong Vocational and Technical College, Hanzhong, 723002 Shaanxi Province China

**Keywords:** Coffin-Siris syndrome, Short stature, Congenital giant nevus, Case report

## Abstract

**Background:**

Coffin-Siris syndrome (CSS) is a rare autosomal dominant disorder characterized by intellectual disability, developmental delay, and characteristic facial features. Few patients with cutaneous phenotype in this rare syndrome have been reported.

**Case presentation:**

Herein, we describe a 12-year-old Chinese girl diagnosed with CSS, who was referred to our hospital because of intellectual disability and short stature. Prominent characteristics of the cutaneous system were observed: (1) A congenital giant nevus from the left frontal and temporal regions to the entire left scalp; and (2) multiple melanocytic nevi on the face and trunk. Whole exome sequencing revealed a novel heterozygous variant in the *ARID1B* gene. Recombinant human growth hormone (rhGH) was given for short stature, and resulted in significantly improved height. No enlargement or malignant transformation of nevi occurred within 4 years of follow-up.

**Conclusion:**

The symptoms in cutaneous system is noteworthy,which may be a neglected phenotype in CSS.The therapeutic response of growth hormone is effective in this patient and no tumor related signs were found.

**Supplementary Information:**

The online version contains supplementary material available at 10.1186/s12887-022-03535-4.

## Background

Coffin-Siris syndrome (CSS) (OMIM#135,900) is a rare autosomal dominant syndrome. Its pathogenesis is related to SWItch/Sucrose Non-Fermentable (SWI/SNF) chromatin remodeling enzymes [1]. To date, mutations in one of the ten SWI/SNF subunits, *ARID1A, ARID1B, SMARCA4, SMARB1, SMARCE1, ARID2, DPF2, SMARCC2, SOX11*, and *PHF6*, have been demonstrated to cause CSS [2]. Approximately 60% of cases were caused by mutations in *ARID1B* [3].

CSS is defined by particular facial traits, developmental delay and mental retardation, and plasia or hypoplasia of the distal phalanx or nail of the fifth and additional digits [4]. Moreover,CSS patients frequently presented with multisystem damage such as cardiac malformations, gastrointestinal and endocrine abnormities, and visual and hearing impairment [4, 5]. Short stature is a common symptom of CSS. However,details on growth hormone (GH) therapy is lacking.Abnormality in cutaneous system have been reported in only three CSS cases so far [6–8]. Among them, two were *ARID1B*-related cases and both presented with vitiligo together with numerous melanocytic nevi [6] or without [7]; and one was *ARID2* deficient case, who presented with anomalous skin pigmentation [8]. Here, we report the clinical characteristics of a short girl re-diagnosed as having *ARID1B*-related CSS, with multiple nevi, and her response to recombinant human growth hormone (rhGH) treatment.

## Case presentation

The patient was the second child of non-consanguineous Chinese parents born at full term. The parents were both healthy.Her birth weight was 2500 g (P50) and length was 49.0 cm (P3). At birth, a black nevus was observed on her left temporal region as well as on her forehead to the entire scalp on the left side, which was not enlarged subsequently. she could not sit until 9-month-old, and walk independently until 18-month-old. She began to speak the words "dada" and "mama" at 24 -month-old and now she can say complete sentences in broken words.At 4 years, she was diagnosed with vitiligo because of asymptomatic multiple leukoplakias on her neck skin. At 7 years, she was diagnosed with attention deficit hyperactivity disorder due to learning difficulties and an intelligence test score of 70. She suffered epileptic attacks since the age of 9 years and presented with generalized tonic–clonic seizures. At the age of 10 years and 9 month, her height was 123 cm (< P3), with bone age at 8 years and 10 month by the Gruelich and Pyle standards. The GH peak on stimulation test was 3.17 ng/mL and insulin-like growth factor 1 level was 136 ng/mL (reference range: 117–771). Thus, she was diagnosed with GH deficiency and started rhGH therapy. The therapy had lasted for 12 months during which rhGH dosage was adjusted to keep within a therapeutic range of 0.12–0.15 mg/kg/day,and her height increased by 13 cm.Insulin-like growth factor-I (IGF-1), blood glucose and thyroid function were followed up every 3 months during the treatment.

**Fig. 1:**
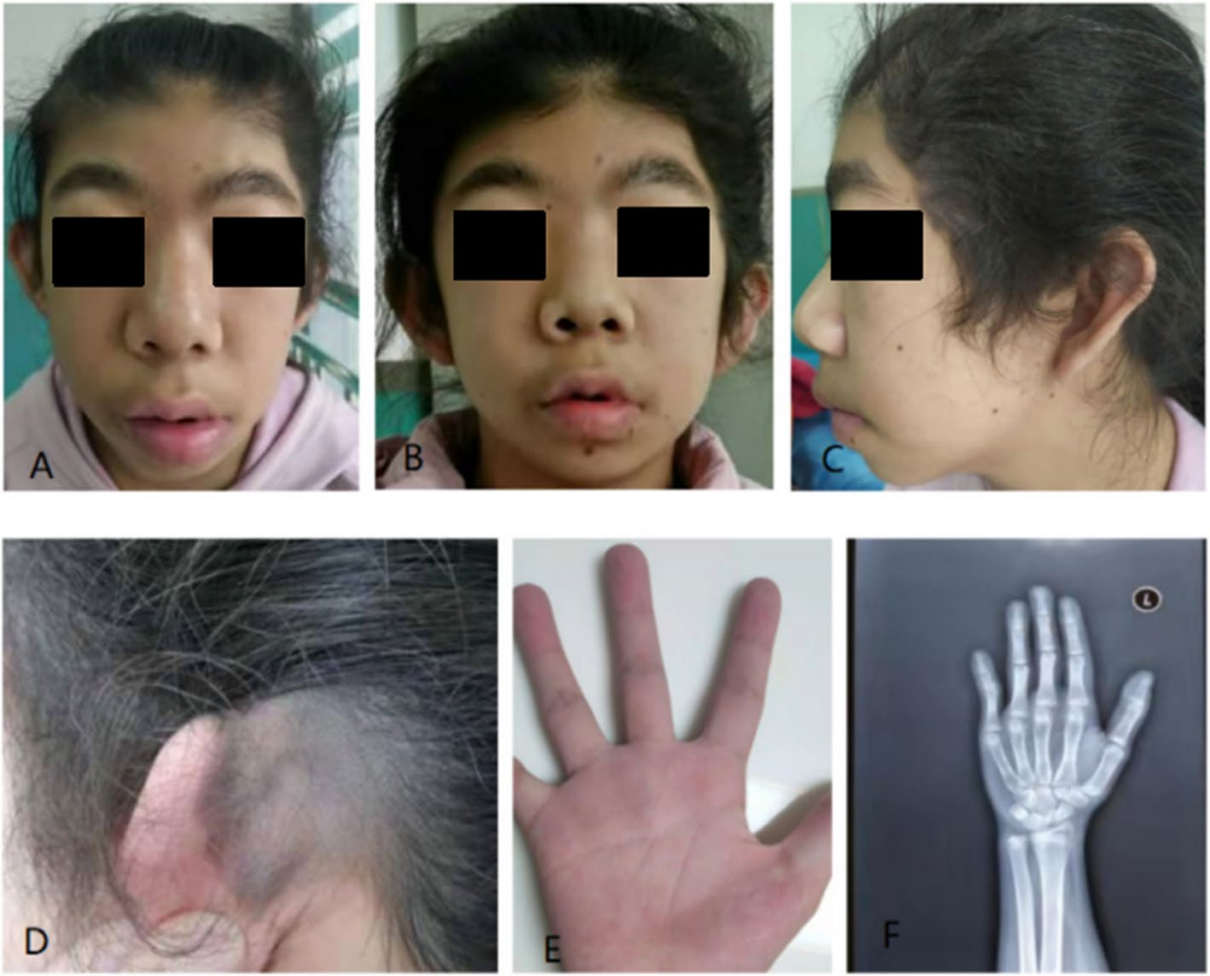
Clinical photographs of a patient with Coffin-Siris syndrome. **A** and **B**: Coarse face, coppery filamentous hair, bushy eyebrows, and wide nose tip; **C**: Small chin, palatal arched elevation, and multiple facial nevi; **D**: Congenital giant nevus on the left scalp; **E**: Single transverse palmar crease; **F**: Short middle phalanx of little finger

**Fig. 2:**
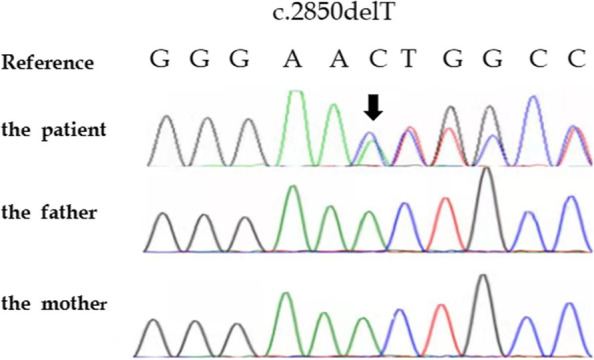
Sanger sequencing results. Sanger sequencing confirmed that the variant NM_001374828.1: c. 3099delT (p.Phe1034Serfs*3) was de novo

She was then referred to our hospital at 12 years due to mental retardation and short stature. Her height was 137 cm (< P3) and weight was 36.5 kg ( P25- P50).On physical examination, distinct features of CSS were noticed (Fig. 1), including: (1) Coarse face, low hairline, bushy eyebrows, broad nasal bridge, high palate, small lower jaw and thick lower lip vermilion, and multiple nevi on the face and trunk; (2) a single transverse palmar crease in the right hand; and (3) dysplasia of the middle segment in the fifth finger showed by radiological examination. Giant nevus on the left scalp was first observed in such case.Laboratory examinations showed no abnormality in routine blood,liver and kidney function, myocardial enzyme,serum electrolytes,tumor marker,and thyroid function tests(supplementary table 1).Cranial magnetic resonance imaging (MRI) and pituitary MRI were normal. Whole exome and Sanger sequencing were performed on blood-extracted DNA from the patient and her father.A heterozygous *ARID1B* variant NM_001374828.1:c.3099delT (p.Phe1034Serfs*3) was identified, and regarded as pathogenic according to the American College of Medical Genetics guidelines [9]. Sanger sequencing confirmed that the variant was de novo (Fig. 2).

The patient was re-diagnosed as having *ARID1B*-related CSS. On demand of the family’s request for cure of short stature, rhGH was administered for 14 months.The girl caught up with a height of 150 cm (P10). No enlargement or malignant transformation of the melanocytic nevi on the face and trunk was observed within 4 years of follow-up.

## Conclusion and discussions

This paper reports for the first time that a congenital giant nevus was present in an *ARID1B*-related CSS case. This case can be explained by a novel heterozygous variant c.3099delT (p.Phe1034Serfs*3). The typical characteristics of facial, hand, little finger, skin, and genetic results promoted us to correct the clinical diagnosis of the affected girl.

Of note, about 80 patients with CSS syndrome due to *ARID1B* mutations have been reported [10]. Yet, multiple nevi and vitiligo were not recorded in OMIM. It is reasonable to recommend that these two phenotypes be considered as characteristics of CSS. Traditional and newly phenotypes of CSS are listed in Table 1.

**Table 1:** Clinical phenotypes of the patient

Part of the body	Phenotype	OMIM	Customary to CSS	Ref
Neuro system	Developmental delay, mental retardation, seizure	√	√	√ [4]
Face	Coarse face, small chin, coppery filamentous hair, bushy eyebrows, wide nose tip	√	√	√ [4]
Skeletal	Dysplasia of the middle segment of the fifth finger	√	√	√ [4]
Stature	Short	√	√	√ [4]
Skin	Melanocytic nevi	—	—	√ [6]
	Congenital giant nevus	—	—	—
	Vitiligo	—	—	√ [6–8]

—: Not recorded in OMIM, not customary to Coffin-Siris syndrome (CSS), and not reported in the public literature

√: Reported in OMIM, customary to CSS, and reported in the public literature

*CSS* Coffin-Siris syndrome

At present, studies have found that approximately 96.0% of patients with *ARID1B* variant show a height of <  − 1 SD and a mean adult height of − 1.8 SD. A few cases mentioned that height improved significantly after rhGH treatment [11], but without detailed description. The patient in this study benefited significantly from rhGH treatment, with height increased 28 cm within 3 years. Her response in height to the treatment is comparable to the GHD patients [12]. Researches in 3 CSS patients suffered from hepatoblastoma [13], schwannoma and meningioma [14], and palillary thyroid carcinoma [15]_._.For the sake of safety, according to the guidelines for the use of growth hormone [12], we have fully screened the patients except for the cases with tumor.

Congenital giant nevus and melanocytic nevi were very prominent in this case. Abnormalities in the Wnt signaling pathway have been demonstrated to be associated with mental retardation in CSS patients [16]. The Wnt signaling pathway interacts with *Gnaq*/*Gnall* and *RAS*, *RAF*, *MEK*, and *ERK* signaling pathways (downstream signaling molecules) to promote the proliferation and differentiation of melanin stem cells and stimulate development of congenital giant nevi [17].Although nevi may be at risk of carcinogenesis,no CSS cases with melanoma have been reported.After the evaluation by dermatologists and oncologists,the giant nevus is likely to be benign.After years of follow-up,no enlargement or increase of nevus was found.

In conclusion,the symptoms in cutaneous system is noteworthy,which may be a neglected phenotype in CSS.The therapeutic response of growth hormone is effective in this patient and no tumor related signs were found.

## Supplementary Information

**Additional file 1. **Serum electrolytes, tumor markers and thyroid function tests.

## Data Availability

The datasets analyzed during the current study are under submission to NCBI ClinVar(The ClinVar assession number: SCV002549918).

## Abbreviations

CSS: Coffin-Siris syndrome
GH: Growth hormone
rhGH: Recombinant human growth hormone
SWI/SNF: SWItch/Sucrose Non-Fermentable
IGF-1: Insulin-like growth factor-I
