# Supplementary material for: Short stature and melanocytic nevi in a girl with ARID1B-related Coffin-Siris syndrome: a case report
Source: BMC Pediatr. 2022 Aug 13;22:486. doi: 10.1186/s12887-022-03535-4 (PMC9375425; doi:10.1186/s12887-022-03535-4)
Supplement: Supplementary file 1 — Additional file 1. Serum electrolytes, tumor markers and thyroid function tests. [file 12887_2022_3535_MOESM1_ESM.doc]

Additional table 1：serum electrolytes, tumor markers and thyroid function tests

| **test items** | test content |
| --- | --- |
| **thyroid function tests** | TSH 3.510(0.72-11.0)uIU/ml  T4 138.00(69.6-219）nmol/L  FT4 24.5(11.5-28.3）pmol/L  T3 1.610 (1.23-4.22）pmol/L  FT3 3.860(3.00-9.28)pmol/L |
| **tumor marker** | CEA 0.953(<5.00ng/ml)  AFP 1.600(<7.0ng/ml)  CA19-9 13.13(<30.00U/ml）  β-hCG <0.100(0-2.60mIU/ml） |
| **serum electrolytes** | K 3.89(3.50-5.30)nmol/L  Na 140.4（137.0-147.0)nmol/L  CL 99.7(99.0-110.0)nmol/L  Ca 2.20(2.11-2.52)nmol/L  IP 1.38(0.85-1.51)nmol/L |
